# Ouabain-Induced Gene Expression Changes in Human iPSC-Derived Neuron Culture Expressing Dopamine and cAMP-Regulated Phosphoprotein 32 and GABA Receptors

**DOI:** 10.3390/brainsci11020203

**Published:** 2021-02-07

**Authors:** Alexander V. Lopachev, Maria A. Lagarkova, Olga S. Lebedeva, Margarita A. Ezhova, Rogneda B. Kazanskaya, Yulia A. Timoshina, Anastasiya V. Khutorova, Evgeny E. Akkuratov, Tatiana N. Fedorova, Raul R. Gainetdinov

**Affiliations:** 1 Laboratory of Clinical and Experimental Neurochemistry, Research Center of Neurology, 125367 Moscow, Russia; july.timoschina@yandex.ru (Y.A.T.); hutorova.anastasiya@mail.ru (A.V.K.); tnf51@bk.ru (T.N.F.); 2 Laboratory of Cell Biology, Federal Research and Clinical Center of Physical-Chemical Medicine Federal Medical Biological Agency, 119435 Moscow, Russia; lagar@rcpcm.org (M.A.L.); oslebedeva@rcpcm.org (O.S.L.); 3 Laboratory of Plant Genomics, Institute for Information Transmission Problems of the Russian Academy of Sciences, 127051 Moscow, Russia; a.fedotova@skoltech.ru; 4 Center of Life Sciences, Skolkovo Institute of Science and Technology, 121205 Moscow, Russia; 5 Biological Department, Saint Petersburg State University, 199034 St. Petersburg, Russia; st059046@student.spbu.ru; 6 Biological Department, Lomonosov Moscow State University, 119991 Moscow, Russia; 7 Department of Applied Physics, Royal Institute of Technology, Science for Life Laboratory, 171 65 Stockholm, Sweden; akkuratov.evgeny@gmail.com; 8 Institute of Translational Biomedicine and Saint Petersburg University Hospital, Saint Petersburg State University, 199034 St. Petersburg, Russia; gainetdinov.raul@gmail.com

**Keywords:** dopamine, GABA, RNA-seq, iPSC, cardiotonic steroids, gene expression, Na^+^,K^+^-ATPase, ouabain, neurons, transcriptome

## Abstract

Cardiotonic steroids (CTS) are specific inhibitors and endogenous ligands of a key enzyme in the CNS—the Na^+^, K^+^-ATPase, which maintains and creates an ion gradient on the plasma membrane of neurons. CTS cause the activation of various signaling cascades and changes in gene expression in neurons and other cell types. It is known that intracerebroventricular injection of cardiotonic steroid ouabain causes mania-like behavior in rodents, in part due to activation of dopamine-related signaling cascades in the dopamine and cAMP-regulated phosphoprotein 32 (DARPP-32) expressing medium spiny neurons in the striatum. Dopaminergic projections in the striatum innervate these GABAergic medium spiny neurons. The objective of this study was to assess changes in the expression of all genes in human iPSC-derived expressing DARPP-32 and GABA receptors neurons under the influence of ouabain. We noted a large number of statistically significant upregulated and downregulated genes after a 16-h incubation with non-toxic concentration (30 nM) of ouabain. These changes in the transcriptional activity were accomplished with activation of MAP-kinase ERK1/2 and transcriptional factor cAMP response element-binding protein (CREB). Thus, it can be concluded that 30 nM ouabain incubated for 16 h with human iPSC-derived expressing DARPP-32 and GABA receptors neurons activates genes associated with neuronal maturation and synapse formation, by increasing the expression of genes associated with translation, vesicular transport, and increased electron transport chain function. At the same time, the expression of genes associated with proliferation, migration, and early development of neurons decreases. These data indicate that non-toxic concentrations of ouabain may induce neuronal maturation, neurite growth, and increased synaptogenesis in dopamine-receptive GABAergic neurons, suggesting formation of plasticity and the establishment of new neuronal junctions.

## 1. Introduction

Over the past few years, our understanding of the physiological role of the Na^+^,K^+^-ATPase and its endogenous regulators, cardiotonic steroids (CTS), in the central nervous system (CNS) has broadened significantly [1,2]. Three isoforms of the Na^+^,K^+^-ATPase catalytic α subunit are present in the brain: α1, which is expressed in all mammal cells, α2, expressed in glial cells and myocytes, and α3, which is neuron-specific in adult organisms [3]. The α3 isoform is responsible for reversing sodium influx which occurs during action potential firing [4]. However, the role of the Na^+^,K^+^-ATPase is not limited to maintaining and restoring the sodium and potassium gradient—it is also involved in regulating various ionotropic and metabotropic receptors, as well as Na^+^-dependent transporters. A number of studies has demonstrated protein-to-protein and functional interaction of the Na^+^,K^+^-ATPase with glutamate NMDA [5,6] and AMPA receptors [7], dopamine D1 and D2 receptors [8,9,10], as well as GABA receptors [11]. Furthermore, the Na^+^ gradient maintained by the Na^+^,K^+^-ATPase facilitates the functioning of the Ca^2+^ transporter (NCX) [12], glycine (GlyT2) [13], glutamate (GLAST и GLT-1) [14] transporters, and others.

At the present moment, CTS, which bind to the Na^+^,K^+^-ATPase catalytic alpha subunit, are the only known specific inhibitors of the enzyme [15,16]. CTS are considered to be endogenous regulators, or hormone-like compounds [1,2,17,18,19]. It is known that CTS can significantly affect dopamine neurotransmission through affecting the function of DAT or dopamine receptors [20,21]. Intracerebroventricular (ICV) CTS injection causes bipolar disorder-like behavior in rodents [21,22], which may be accompanied by neuron damage [22,23]. In the amphetamine mania model in rats it was shown that intracerebroventricular injection of CTS antibodies neutralizes the effects of amphetamine administration [24] which means that not only exogenous but also endogenous CTS may contribute to mania-like behavior. As we have previously shown, ICV injection of ouabain in mice causes mania-like behavior, decreased dopamine reuptake, and an increase in phosphorylation of ERK1/2, Akt, and GSK3β in striatal tissue [21]. Alterations in behavior and activity of signaling cascades were significantly affected by administration of D2 dopamine receptor antagonist haloperidol [21]. In the striatum, dopaminergic projections innervate GABAergic medium spiny neurons which express D1- and D2-like dopamine receptors and an integrator of dopamine neurotransmission dopamine and cAMP-regulated phosphoprotein 32 (DARPP-32) [25,26,27]. It is also known that synergistically interacting dopamine D1 and NMDA receptors mediate non-vesicular transporter-dependent GABA release from rat striatal medium spiny neurons [11]. In light of the knowledge of reciprocal circuit of regulation between dopaminergic neurons in substantia nigra and striatal GABAergic neurons [28], the question of what long-term effects CTS may have on dopamine-receptive medium spiny GABA neurons arises.

The effects of CTS, mediated both by interaction with partner proteins and by changes in the ion gradient, are enacted through the activation of intracellular signaling pathways, such as MAP-kinase (ERK1/2, p38, JNK) [29], IP3K, PKC, and Akt [30,31,32,33,34,35]. These signaling pathways, in turn, can affect both properties of various proteins via phosphorylation, and the expression of genes via the activation of various transcription factors. As such, the investigation of the effects of CTS on gene expression is key for understanding the mechanisms underlying the response they evoke in cells. Ouabain-induced gene expression (c-fos and c-jun) was first described in 1996 in cardiomyocytes [36]. Furthermore, it was shown that ouabain and marinobufogenin cause changes in expression of genes associated with translation regulation [37]. However, HUVEC cells have a different gene expression profile than neuron cells, and lack Na^+^,K^+^-ATPase α2 and α3 subunits, which precludes extrapolation of acquired data onto neurons. It should also be noted that the study in question evaluated CTS-induced changes in expression of select gene groups using GeneChip. A transcriptome analysis of ouabain-induced expression changes in all genes expressed in human neurons would reflect the full spectrum of processes influenced by CTS. Therefore, the goal of this study was to evaluate the effect of non-toxic concentration of ouabain on gene expression profiles of human iPSC-derived neurons expressing DARPP-32 and GABA receptors.

## 2. Materials and Methods

### 2.1. Cultivation and Differentiation of iPSC

Experiments concerning cell cultures and media composition were conducted on a neuron culture derived from human iPSC in accordance with existing protocols [38]. The iPSC were derived from fibroblasts taken from a healthy donor. iPSC were cultivated in a mTeSR1 medium (Stemcell Technologies, Vancouver, BC, Canada), on Matrigel™ substrate (BD Biosciences, San Jose, CA, USA). Cells were passaged with 0.05% Trypsin (Invitrogen, Carlsbad, CA, USA) and cryoconservated in mFreSR1 medium (Stemcell Technologies). iPSC were cultivated in mTeSR1 medium on Matrigel until 80–90% confluence, after which the medium was replaced with a mixture of 1:4 mTeSR1 and K-1 medium for two days. The cells were then cultivated in K-1 medium over the course of 5–7 days, and in K-2 medium for 7–9 more days. Next, K-2 medium was replaced with K-3, in which the cells were held until the formation of neuronal rosettes. Neuronal progenitors were cultivated by overgrowing and dissociating cells with the help of StemPro Accutase Cell Dissociation Reagent (Thermo Scientific, Waltham, MA, USA), after which cells were held in K-3 medium until the fourth passage. During this time, neuronal progenitors were incubated in K-4 medium over the course of 10 days until maturation.

Twenty-four hours prior to experimental procedures K-4 medium was substituted with Neurobasal A (Thermo Scientific with the addition of 5% B27 (Thermo Scientific). All further experiments were conducted on neurons cultivated using the same protocol, with a prior evaluation of neuronal differentiation.

### 2.2. Immunocytochemical Confirmation of iPSC Differentiation into Neurons

To prepare for immunocytochemical staining, the cultural medium was removed, after which the cultures were rinsed with PBS two times. Cells were then fixated with 4% paraformaldehyde for 10 min at room temperature. After fixation, cells were incubated with 0.1% TRITON X-100 in PBS, and blocked with 1% BSA in PBS with 0.1 TWEEN 20.

Cells were then incubated at room temperature with primary antibodies diluted at a ratio of 1:500 in PBS with 0.1% TWEEN 20. The following primary antibodies were used: β3 tubulin (mouse) (Abcam, Cambridge, MA, USA), GFAP (rabbit) (AbCam), MAP2 (mouse) (AbCam), DARPP-32 (rabbit) (Abcam), and Th (rabbit) (AbCam). After incubation with the primary antibodies, cells were rinsed with 1% BSA in PBS with 0.1% TWEEN 20. Following rinsing, cells were incubated with secondary anti-mouse and anti-rabbit antibodies tagged with fluorescent markers over the course of 1 h. Prior to fluorescent microscopy (Nikon, Tokyo, Japan), a solution of the nuclear staining agent DAPI in PBS was added to the cells.

### 2.3. Western Blot

Following incubation with ouabain, cells were washed twice with cold HBSS and lysed in RIPA buffer (Sigma, St. Louis, MO, USA), containing cocktails of protease and phosphatase inhibitors (Sigma). The lysates were clarified by centrifugation at 12,000 × *g* for 10 min. Protein concentration in the samples was measured using DC Protein Assay Kit (Bio-Rad, Hercules, CA, USA). Samples were subjected to 12% SDS-PAGE, transferred to a PVDF membrane and probed with appropriate antibodies by standard procedures. The following primary and secondary antibodies were used: p-ERK1/2 (Thr202/Tyr204, Santa Cruz Biotechnology, Dallas, TX, USA, Cat# sc-136521, RRID:AB_10856869); ERK1/2 (Santa Cruz Biotechnology, cat no. sc-135900, RRID:AB_2141283); β-Actin (Cell Signaling Technology, Danvers, MA, USA, cat no. 8457, RRID:AB_10950489); α1 (DSHB, Iowa City, IA, USA); α3 (Thermo Scientific); anti-rabbit IgG-HRP (Cell Signaling Technology, cat no. 7074, RRID:AB_2099233); anti-mouse IgG-HRP (Cell Signaling Technology, cat no. 7076, RRID:AB_330924). Immunoreactive bands were detected using either SuperSignal West Femto Maximum Sensitivity Substrate or SuperSignal West Pico Chemiluminescent Substrate (Thermo Scientific). Chemiluminescence was detected using ChemiDoc XRS+ system (Bio-Rad); intensity was counted using Image Lab 3.0 software (Bio-Rad). The level of phosphorylation was counted as the ratio between signals from phosphorylated and total forms of ERK1/2. β-actin was used as the reference for pCREB and as loading control to ensure that the total amount of kinases did not change. Statistical analysis of the data was performed using GraphPad Prism 7 software. Data analyses of multiple groups with two variables was performed using Shapiro–Wilk normality test and one-way ANOVA, *p* value was calculated using Dunnett’s multiple comparisons test. The q value, or the difference between the two means divided by the standard error of that difference computed from all the data, was also calculated using Dunnett’s test. N—samples per group, the number of wells in the 12-well cell plate used in the measurement for each group.

### 2.4. MTT Assay

Cell viability was evaluated using MTT assay in 96-well plates. The method is based on the reduction of yellow 3-(4,5-dimethyl-2-thiazolyl)-2,5-diphenyl-2H-tetrazolium bromide (MTT) by living cells to blue formazan. The procedure was carried out as described in [29]. Sample absorbance was measured using a Synergy H4 plate reader (BioTek, Winooski, VT, USA). Data is presented as a percentage of the signal in control wells with intact cells. Data analyses of multiple groups with two variables was performed using Shapiro–Wilk normality test and one-way ANOVA, *p* value was calculated using Sidak’s multiple comparisons test. The t value, or the calculated difference represented in units of standard error, was also calculated using Sidak’s multiple comparisons test. N—samples per group, the number of wells of the 96-well cell plate used in the measurement for each group.

### 2.5. Transcriptome Analysis of Neuron Culture Derived from Human iPSC after 16 h Incubation with 30 nM Ouabain

To investigate the effects of 30 nM ouabain on the transcriptome of human iPSC-derived neurons, cells were cultivated in 12-well plates. Ouabain in a final concentration of 30 nM was added to the medium, and the cells were incubated for 16 h. RNA was then extracted from the cultures using RNeasy Plus (Quiagen, Germantown, MD, USA) and stored at −70 °C. The concentration of RNA in each sample was measured using a Qubit v.1 fluorimeter and an RNA Broad range reagent set (Thermo Fisher, Waltham, MA, USA). Samples were then diluted to a concentration of 3 ng/mL and analyzed using capillary electrophoresis on a Bioanalyzer2100 (Agilent, Santa Clara, CA, USA) and an RNA Pico 6000 reagent set (Agilent Technologies, Santa Clara, CA, USA). RNA integrity number (RIN) based on the ratio of 18S and 28S ribosomal RNA was deduced for each sample [39]. Since the standard protocol of RNA sample preparation relies on sequestration of polyadenylated transcripts using magnetic particles associated with olygo-dT, RNA with a RIN no less than 7 is recommended [40]. All acquired samples fulfilled this requirement.

Samples were prepared for sequencing using the NEBNext Ultra II RNA reagent set (New England Biolabs, Ipswich, MA, USA). Sequencing was conducted using the NextSeq500 (Illumina, San Diego, CA, USA) sequenator in single-reads with 75 nucleotide read length. After sequencing completion, demultiplexination (mapping of sequenced sequences to samples using the assigned indexes) of acquired data and conversion to fastq format using bcl2fastq2 software (Illumina) was performed.

### 2.6. Bioinformatics Analysis

Primary quality control was performed using FastQC software (https://www.bioinformatics.babraham.ac.uk/projects/fastqc/, accessed on 20 December 2019). It was shown that all samples are optimal on most parameters, excluding nucleotide composition in the beginning of each read, which shows significant deviation from random variables. This is caused by differential primer binding affinity to RNA during reverse transcription, which causes inconsistency in representation of nucleotides near 5′ ends of the fragments. This is typical for all transcriptome libraries assembled from reverse transcription from random primers, independent of the organism or tissue from which RNA is extracted [41], and does not interfere with further analysis.

Trimming from adapters and falsely read positions was performed using CLC Genomics Workbench 7.0.3 with the following parameters: Adapter trimming = Illumina Truseq, maximum number of unrecognized nucleotides = 1, cut-off point for quality level = 0.01 (corresponds to Q20 quality level), cut-off point for read length = 25 nucleotides. Trimmed reads were mapped to the genome version GRCh38 using CLC Genomics Workbench 7.0.3 with the following parameters: mapping only to coding segments, only uniquely mapped reads are counted, level of similarity = 97%, portion of mapped segment = 100%.

Differentially expressed genes were found using the DESeq2 package in the R statistical environment [42]. Differential gene expression was considered significant for *p*-value corrected for multiple testing (FDR) less than 0.05 and fold change (mean expression in the experimental group divided by the mean expression in the control group) greater or equal to 1.2.

### 2.7. Enrichment and Gene Ontology (GO) Annotation

All GO Annotation, KEGG enrichment analysis, and clusterization was carried out using the DAVID functional annotation tools [43]. CirGO software [44] was used to visualize the results of GO clusterization. EnhancedVolcano was used to represent the cut-off point for fold change in gene expression (1.2-fold change was taken as the minimum significant value, in accordance with other publications) [45,46]. MAGIC software [47] with the 5 kb gene matrix was used to predict possible transcription factors.

## 3. Results

### 3.1. Confirmation of Neuronal Differentiation of the iPSC Culture

To confirm neuronal differentiation of the iPSC culture, colonies from the same passage as the ones used during the rest of the experiments presented in this work were stained with antibodies to β3 tubulin, Microtubule Associated Protein 2 (MAP2), Dopamine and CAMP-Regulated Neuronal Phosphoprotein 32 (DARPP-32), and Glial Fibrillary Acidic Protein (GFAP) и tyrosine hydroxylase (Th).

**Figure 1:**
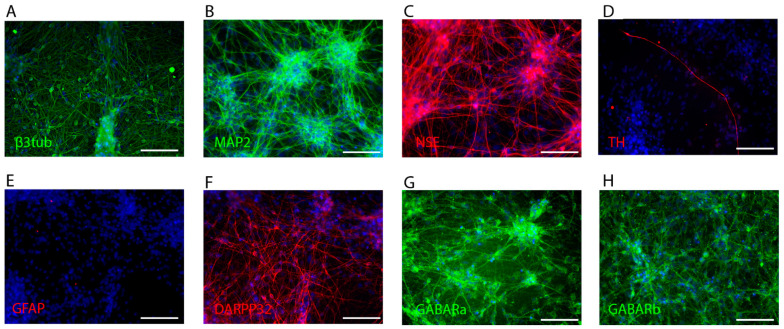
Immunocytochemical staining of human iPSC-derived neuron culture. (**A**) β3 tubulin (green), (**B**) MAP2 (green), (**C**) NSE (red), (**D**) TH (red), (**E**) GFAP (red), (**F**) DARPP-32 (red), (**G**) GABARa (green), and (**H**) GABARb (green). All images include DAPI staining (blue). Scale bar for all images is 100 μm.

As seen in Figure 1, the cell cultures were positive for NSE, β3 tubulin, and MAP2, which are general neuronal differentiation markers, DARPP-32, GABARa, and GABARb—markers of GABAergic striatal neurons, and trace amounts of GFAP. At the same time, there are few Th-positive neurons. As such, we draw the conclusion that the derived culture population is enriched with GABAergic, but not dopaminergic neurons. A relatively small quantity of cells expressing a glial phenotype is also present.

One important characteristic of neurons is the expression of the α3 isoform of the Na^+^,K^+^-ATPase α-subunit. Western blot was used to demonstrate that the human iPSC-derived neuron culture used in these experiments expresses both α3 and α1 isoforms of the Na^+^,K^+^-ATPase α-subunit. 4 h incubation with 3 nM, 30 nM, and 300 nM ouabain does not noticeably affect the expression of either of these proteins (Figure S1).

### 3.2. Evaluation of the Effects of 48 h Incubation with a Range of Ouabain Concentrations on Neuron Viability

To evaluate the effects of ouabain on viability of the neurons culture, toxicity of ouabain in a concentration range of 3 nM to 10 nM was assessed. The MTT-test was used to evaluate viability of the culture post 48 h of incubation with ouabain.

**Figure 2:**
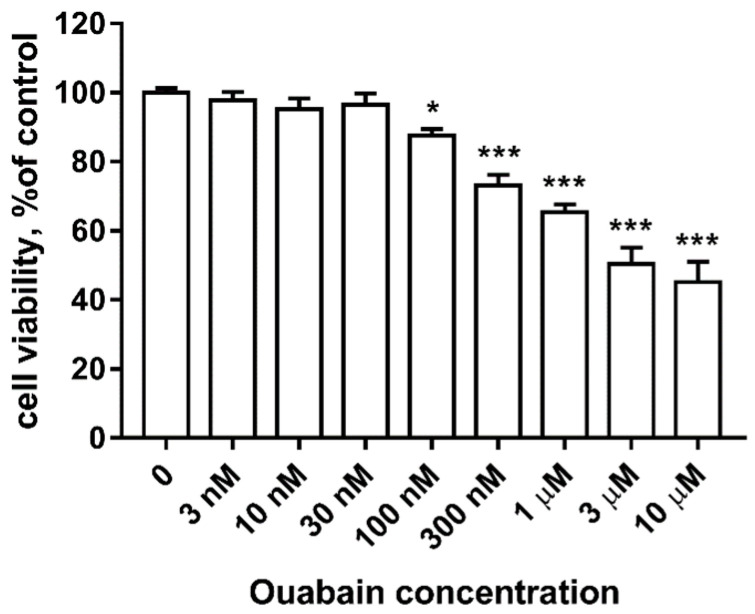
Effects of 48 h incubation with 3 nM to 10 mkM ouabain on human iPSC-derived neuron culture viability according to the MTT-test. Data is presented in percent mean ± SEM, with intact culture taken as 100%. *n* = 12. * *p* < 0.05, *** *p* < 0.001.

As seen in Figure 2, ouabain causes a dose-dependent decrease in culture viability (*p* < 0.0001, F = 43.34). At the same time, ouabain at concentrations of 3 nM, 10 nM, and 30 nM did not affect culture viability. The minimal toxic concentration of ouabain was found to equal 100 nM, which caused a decrease in culture viability to 87.65 ± 1.86% of the control (*p* = 0.048, t = 2.811). 300 nM ouabain caused a decrease in culture viability to 73.14 ± 3.08% (*p* < 0.0001, t = 6.723), 1 mkM ouabain to 65.35 ± 2.25% (*p* < 0.0001, t = 8.882), 3 mkM ouabain to 50.4 ± 4.79% (*p* < 0.0001, t = 11.29), and 10 mkM ouabain to 45.1 ± 5.94% (*p* < 0.0001, t = 12.49). As such, 30 nM ouabain was found to be the maximum non-toxic concentration among the investigated range.

### 3.3. Evaluation of the Effects of 4 h Culture Incubation with Ouabain on ERK1/2 and CREB Activation

In order to investigate the effects of chosen ouabain concentrations on the activation of intracellular signaling cascades in human iPSC-derived neuron culture, the effects of 3 nM, 30 nM, and 300 nM ouabain on ERK1/2 and CREB activation were evaluated. Activation was evaluated using western blot of culture lysates, which showed the level of phosphorylated (pERK1/2), total ERK1/2, and CREB post a 4 h incubation with ouabain. β-actin was used as reference to evaluate the intensity of CREB phospo-forms, while ERK1/2 total was used as reference for pERK1/2 band intensity.

**Figure 3:**
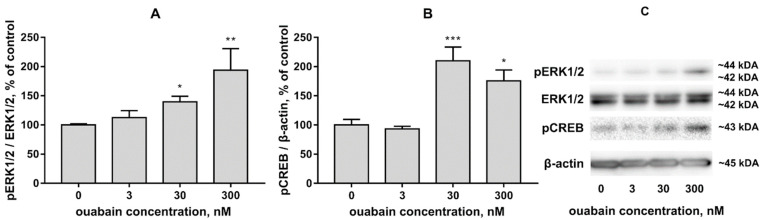
The effects of 4 h culture incubation with 3 nM, 30 nM, and 300 nM ouabain on ERK1/2 (**A**) and CREB (**B**) activation. Data is presented as percent mean ± SEM, where intact culture values are taken as 100%. *n* = 6. Representative immunoreactive bands (**C**). * *p* < 0.05, ** *p* < 0.01, *** *p* < 0.001.

According to the acquired data, 4 h culture incubation with ouabain leads to an increase in ERK1/2 activation (*p* < 0.0001, F = 9.61). While the increase in ERK1/2 activation induced by incubation with 3 nM ouabain was not statistically significant (*p* = 0.83, q = 0.6895), 30 nM ouabain caused ERK1/2 activation to increase to 143.6 ± 9.88% (*p* = 0.048, q = 2.31), and 300 nM ouabain—to 194 ± 36.86% (*p* = 0.0057, q = 3.32) Figure 3A,C. Similarly, 4 h culture incubation with ouabain induced CREB activation (*p* = 0.0021, F = 10.27). Incubation with 3 nM ouabain did not cause a statistically significant change in CREB activation (*p* = 0.982, q = 0.295). 30 nM ouabain caused an increase to 209.8 ± 23.56% in CREB activation (*p* = 0.0003, q = 5.05), and 300 nM caused an increase to 175.4 ± 18.86% (*p* = 0.0172, q = 3.1) Figure 3B,C. As such, we demonstrated that 4 h culture incubation with ouabain in a concentration of 30 nM and greater causes activation of both ERK1/2 and CREB.

### 3.4. Acquisition of Differentially Expressed Genes (DEGs) Set for Further Analysis

After preliminary RNA-seq data quality control and analysis, the DEseq2 R package was used to analyze and sort DEGs detected post 16 h incubation of human iPSC-derived neurons with 30 nM ouabain. 479 genes were found to have different expression levels in the control and ouabain-affected cultures (FRD < 0.05). 345 genes among them were upregulated, while 134 were downregulated. In addition to the results of immunocytochemical staining, according to RNAseq data, the cultures used in the experiment express markers of GABAergic medium spiny neurons, such as glutamate decarboxylase (RPKM = 1.36) and dopamine D1 (RPKM = 0.14), D2 (RPKM = 0.062) and D4 (RPKM = 1.3) receptors.

**Figure 4:**
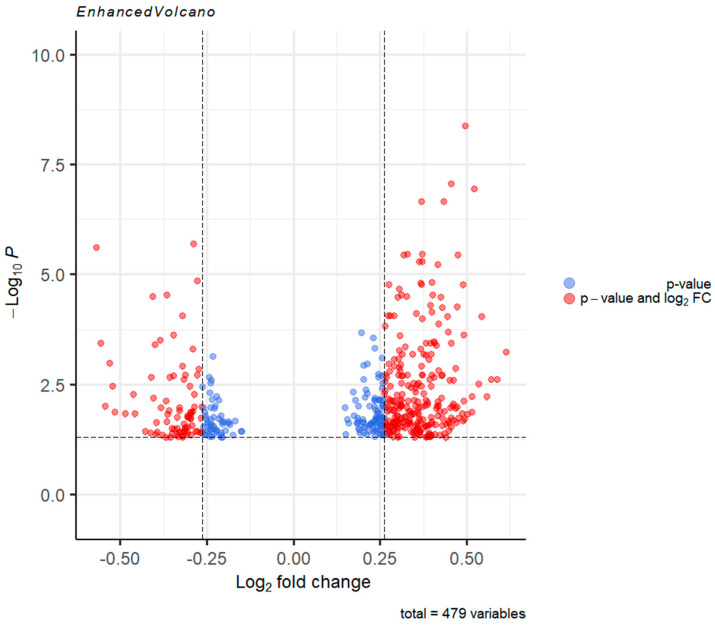
Volcano plot representing the statistically significant DEGs. Genes below the *p*-value cut-off were omitted from the plot. Genes which displayed fold change <1.2 are colored blue. Genes with fold change ≥1.2 are shown in red. Data plotted using the EnhancedVolcano software package Bioconductor version: Release (3.12). R package version 1.8.0. R version 4.0.3.

The resulting DEGs were filtered by fold change—only genes whose expression change was greater than or equal to 1.2 fold were selected for further analysis. The resulting lists consisted of 257 statistically significant upregulated genes, and 81 statistically significant downregulated genes (Figure 4).

### 3.5. Evaluation of DEGs Significance Based on Tissue Specificity of Expression

Using the online DAVID functional annotation tools (UP_TISSUE), we performed an analysis of the two resulting lists—downregulated and upregulated genes. According to the analysis most of the DEGs are brain-specific. This is the case for both the downregulated (Count = 57; *p*-Value = 4.65 × 10^−8^; Fold Enrichment = 1.72) and upregulated (Count = 132; *p*-Value = 3.09 × 10^−5^ Fold Enrichment = 1.31) genes (Supplementary materials, sheet “DEG list (filtered by padj)”).

### 3.6. Description of the Main GO Groups Containing Upregulated Genes

**Figure 5:**
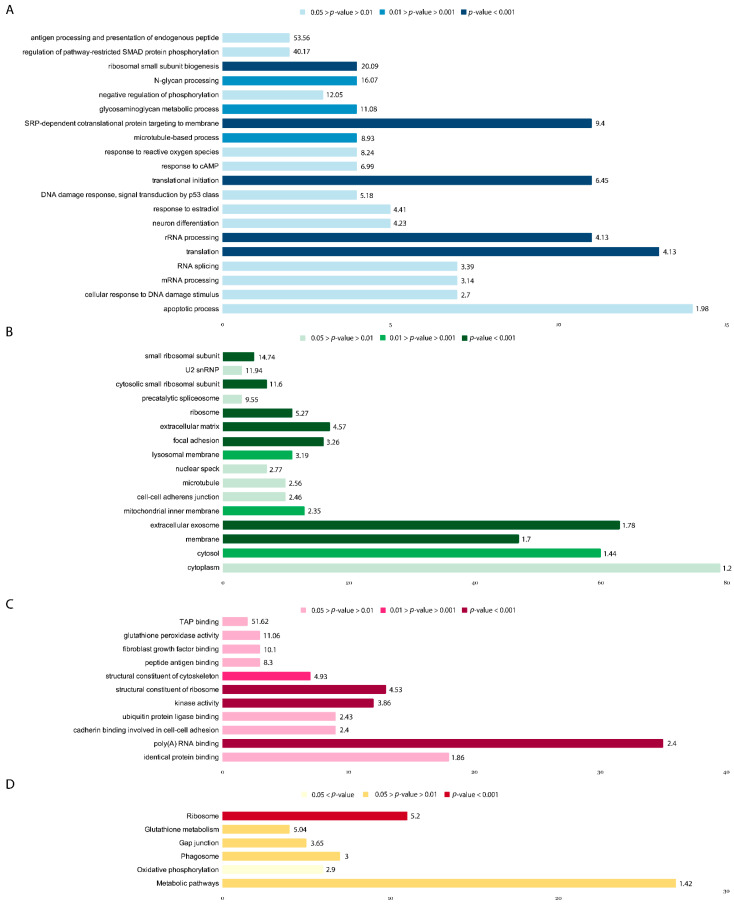
Upregulated GO groups by biological processes (**A**); Upregulated GO groups by cellular components (**B**); Upregulated GO groups by molecular function (**C**); Upregulated gene groups by KEGG (**D**). Labels for each GO group are plotted on the *y*-axis, the number of genes present within the GO group—on the *x*-axis. Fold enrichment scores for the corresponding GO groups are presented at the end of each bar. Results within a given plot are color-coded by *p*-value.

As mentioned previously, 256 genes, the expression of which significantly (*p* < 0.05) increased more than 1.2 fold in comparison to the control culture after a 16 h incubation with 30 nM ouabain, were selected for further analysis. The selected genes were sorted into gene ontology (GO) groups in accordance with the three ontologies: Biological processes (GOTERM_BP_DIRECT)—44 groups (Groups of interest shown in Figure 5A, Supplementary Table S1), cellular components (GOTERM_CC_DIRECT)—29 groups (Figure 5B, Supplementary Table S2), molecular function (GOTERM_MF_DIRECT)—20 groups (Groups of interest shown in Figure 5C, Supplementary Table S3). Genes were also grouped in accordance with their involvement in metabolic pathways using the KEGG database (KEGG_PATHWAY)—7 groups (Groups of interest shown in Figure 5D, Supplementary Table S4). Two genes associated with synapse organization also should be mentioned separately: AGRN—Agrin Proteoglycan, and ELFN1—Extracellular Leucine Rich Repeat and Fibronectin Type III Domain Containing 1. The full lists of genes and GO groups can be found in Supplementary Materials, sheet “UPreg genes”.

Among the groups of genes sorted in accordance with biological processes (Figure 5A), the following groups should be mentioned: GO:0000302~response to reactive oxygen species (4 genes: PTPRN—Protein Tyrosine Phosphatase Receptor Type N, PRDX5—Peroxiredoxin-5, GSTP1—Glutathione S-Transferase Pi 1, GPX1—Glutathione Peroxidase 1; *p* = 0.012, Fold Enrichment = 8.24); GO:0051591~response to cAMP (4 genes: BSG—Basigin, VGF—VGF Nerve Growth Factor Inducible, JUND—Transcription factor JunD, PTPRN; *p* = 0.019, Fold Enrichment = 6.98); GO:0006915~apoptotic process (14 genes: PUF60—Poly(U) Binding Splicing Factor 60, TRAF4—TNF Receptor Associated Factor 4, MFSD10—Major facilitator superfamily domain-containing protein 10, FKBP8—FKBP Prolyl Isomerase 8, MAP3K10—Mitogen-Activated Protein Kinase Kinase Kinase 10, PIDD1—P53-Induced Death Domain Protein 1, SCAND1—SCAN box domain-containing protein, SQSTM1—Sequestosome 1, ADAM15—ADAM Metallopeptidase Domain 15, CST3—Cystatin C, SLC25A6—Adenine Nucleotide Translocator 3, MRPL41—Mitochondrial Ribosomal Protein L41, PRDX5, MAPK3—Mitogen-Activated Protein Kinase 3; *p* = 0.024, Fold Enrichment = 1.98); GO:0042326~negative regulation of phosphorylation (3 genes: CDKN1C—Cyclin Dependent Kinase Inhibitor 1C, BMP7—Bone Morphogenetic Protein 7, CDKN1A—Cyclin Dependent Kinase Inhibitor 1A; *p* = 0.025, Fold Enrichment = 12.05); GO:0030182~neuron differentiation (5 genes: BRSK2—BR Serine/Threonine Kinase 2, ITM2C—Integral Membrane Protein 2C, BRSK1—BR Serine/Threonine Kinase 1, PIGT—Phosphatidylinositol Glycan Anchor Biosynthesis Class T, PIN1—Peptidyl-prolyl cis-trans isomerase NIMA-interacting 1; *p* = 0.03, Fold Enrichment = 4.23). The full list of genes and GO groups can be found in Supplementary Table S1.

GO sorting DEGs upregulated in the presence of 30 nM ouabain according to cellular components (Figure 5B) yielded the following groups: GO:0031012~extracellular matrix (17 genes: FGFBP3, PLEC, CRIP2, VIM, FLNA, COL6A1, SLC25A6, AGRN, RPS16, RPL30, MMP15, TUBB4B, LTBP4, RPS19, BMP7, ADAMTS10, RPS11; *p* = 1.06 × 10^−6^, Fold Enrichment = 4.57); GO:0070062~extracellular exosome (63 genes: BSG, SDF4, CD63, ATP5MG, GSTP1, TUBB2A, PAFAH1B3, RPS9, CST3, TTYH3, RPS16, TUBB4B, TMEM8A, SERPINH1, LTBP4, SLC7A5, GPX1, MAPK3, TUBGCP6, FTL, FLOT2, SLC9A3, PSMA7, ACTN4, PFKL, UBE2M, CD81, PHLDA3, ARMC9, ALDOA, CKB, OTUB1, FSCN1, RPS11, IGSF8, RPL23A, MTA1, VIM, IGFBP2, GPX4, ADAM15, PCSK1N, RPL30, SNRPD2, RPS19, PRDX5, SLC44A2, ITM2C, TUBB4A, ENTPD6, PLEC, GAA, PLXNB2, TRMT112, FLNA, HLA-C, COL6A1, SQSTM1, HLA-A, SLC3A2, AGRN, ELFN1, GPC1; *p* = 3.30 × 10^−6^, Fold Enrichment = 1.78); GO:0022627~cytosolic small ribosomal subunit (7 genes: FAU, RPS9, RPS24, RPS16, RPS19, RPS15, RPS11; *p* = 2.84 × 10^−5^, Fold Enrichment = 11.6); GO:0005840~ribosome (11 genes: FAU, RPL23A, RPS9, RPS24, RPS16, RPL30, RPL18, RPS19, RPS15, RPL13, RPS11; *p* = 4.68 × 10^−5^, Fold Enrichment = 5.27); GO:0005925~focal adhesion (16 genes: BSG, FLOT2, ACTN4, PLEC, CD81, VIM, RPS15, PDLIM7, FLNA, RPS9, RPS16, RPL30, RPL18, RPS19, MAPK3, RPS11; *p* = 1.27 × 10^−4^, Fold Enrichment = 3.26); GO:0016020~membrane (47 genes; *p* = 3.00 × 10^−4^, Fold Enrichment = 1.7); GO:0015935~small ribosomal subunit (5 genes: FAU, RPS9, RPS24, RPS16, RPS15; *p* = 3.34 × 10^−4^, Fold Enrichment = 14.74); GO:0005829~cytosol (60 genes: *p* = 0.0023, Fold Enrichment = 1.44); GO:0005765~lysosomal membrane (11 genes: COL6A1, SLC44A2, CD63, AP3D1, TMEM175, ITM2C, SPNS1, GAA, TMEM8A, SPPL2B, ABCA2; *p* = 0.0024, Fold Enrichment = 3.19); GO:0005743~mitochondrial inner membrane (13 genes: ATP5MG, SPNS1, NDUFB7, AURKAIP1, NDUFS7, NDUFS5, MRPS26, SLC25A6, MRPL41, NME4, CYC1, ACADVL, TIMM44; *p* = 0.0098, Fold Enrichment = 2.35); GO:0005874~microtubule (10 genes: TUBB2B, MTA1, TUBB4A, TUBB4B, KIFC2, TUBB2A, TBCB, TUBGCP6, CDK5RAP3, CAMSAP3; *p* = 0.0167, Fold Enrichment = 2.56); GO:0005913~cell-cell adherens junction (10 genes: BSG, FLNA, FLOT2, PUF60, ALDOA, RPL23A, SLC3A2, PLEC, FSCN1, H1FX; *p* = 0.021, Fold Enrichment = 2.46); GO:0005686~U2 snRNP (3 genes: SF3B5, SF3A2, SNRPD2; *p* = 0.0255, Fold Enrichment = 11.94); GO:0005737~cytoplasm (79 genes: *p* = 0.038, Fold Enrichment = 1.2); GO:0071011~precatalytic spliceosome (3 genes: SF3B5, SNRNP70, SNRPD2; *p* = 0.0387, Fold Enrichment = 9.55), GO:0016607~nuclear speck (7 genes: RING1, SNRNP70, CBX4, SF3A2, AKAP8L, AKAP17A, PIN1; *p* = 0.041, Fold Enrichment = 2.77). The full list of genes and GO groups can be found in Supplementary Table S2.

DEGs upregulated in the presence of 30 nM ouabain into GO groups by molecular function (Figure 5C) can be found in Supplementary Table S3. Among them are the following: GO:0044822~poly(A) RNA binding (35 genes: *p* < 0.001, Fold Enrichment = 2.4); GO:0003735~structural constituent of ribosome (13 genes: RPL23A, RPS24, RPS15, FAU, RPS9, SLC25A6, RPS16, RPL30, MRPL41, RPL18, RPS19, RPL13, RPS11; *p* < 0.001, Fold Enrichment = 4.53); GO:0016301~kinase activity (12 genes: BRSK2, CKB, OBSL1, CDKN1C, MAPK8IP3, AKAP8L, TAOK3, PRKCSH, MAPK3, CDKN1A, AURKAIP1, DGKZ; *p* < 0.001, Fold Enrichment = 3.86); GO:0005200~structural constituent of cytoskeleton (7 genes: TUBB2B, TUBB4A, AGRN, TUBB4B, VIM, TUBB2A, TUBGCP6; *p* = 0.003, Fold Enrichment = 4.93); GO:0042802~identical protein binding (18 genes: PUF60, TRAF4, SDF4, PSMA7, RABAC1, FKBP8, PFKL, SSNA1, SRM, DRAP1, VIM, SCAND1, PAFAH1B3, ALDOA, SQSTM1, CST3, PRMT1, FTL; *p* = 0.017, Fold Enrichment = 1.86); GO:0004602~glutathione peroxidase activity (3 genes: GPX4, GSTP1, GPX1; *p* = 0.029, Fold Enrichment = 11.06); GO:0031625~ubiquitin protein ligase binding (9 genes: TRAF4, CDC34, SQSTM1, CKB, UBE2M, OTUB1, ANAPC2, UBXN1, CDKN1A; *p* = 0.032, Fold Enrichment = 2.43); GO:0098641~cadherin binding involved in cell-cell adhesion (9 genes: BSG, FLNA, PUF60, ALDOA, RPL23A, SLC3A2, PLEC, FSCN1, H1-10; *p* = 0.034, Fold Enrichment = 2.4); GO:0017134~fibroblast growth factor binding (3 genes: FGFBP3, GPC1, RPS19; *p* = 0.035, Fold Enrichment = 10.1); GO:0046977~TAP binding (2 genes: HLA-C, HLA-A; *p* = 0.038, Fold Enrichment = 51.62); GO:0042605~peptide antigen binding (3 genes: HLA-C, HLA-A, SLC7A5; *p* = 0.049, Fold Enrichment = 8.29).

Sorting DEGs upregulated in the presence of 30 nM ouabain by KEGG pathway (Figure 5D) yielded the following groups: hsa03010:Ribosome (11 genes: FAU, RPL23A, RPS9, RPS24, RPS16, RPL30, RPL18, RPS19, RPS15, RPL13, RPS11; *p* = 4.16 × 10^−5^, Fold Enrichment = 5.2); hsa04145:Phagosome (7 genes: HLA-C, ATP6V0B, TUBB2B, HLA-A, TUBB4A, TUBB4B, TUBB2A; *p* = 0.028, Fold Enrichment = 3); hsa00480:Glutathione metabolism (4 genes: GPX4, SRM, GSTP1, GPX1; *p* = 0.043, Fold Enrichment = 5.04); hsa04540:Gap junction (5 genes: TUBB2B, TUBB4A, TUBB4B, TUBB2A, MAPK3; *p* = 0.046, Fold Enrichment = 3.65); hsa01100:Metabolic pathways (ISYNA1, SRM, INPP5E, ATP5MG, PNPLA2, MAN2A2, DGKZ, ATP6V0B, PAFAH1B3, PIGQ, NDUFS5, MVD, PIGT, ACADVL, GUK1, B3GALT6, PFKL, GAA, NDUFB7, MAN1B1, NDUFS7, ALDOA, CKB, PTGES2, MGAT4B, NME4, CYC1; *p* = 0.047, Fold Enrichment = 1.42); hsa00190:Oxidative phosphorylation (6 genes: ATP6V0B, NDUFS7, NDUFS5, ATP5MG, NDUFB7, CYC1; *p* = 0.054, Fold Enrichment = 2.9). The full list of genes and KEGG groups can be found in Supplementary Table S4.

### 3.7. Description of the Main GO Groups Containing Downregulated Genes

**Figure 6:**
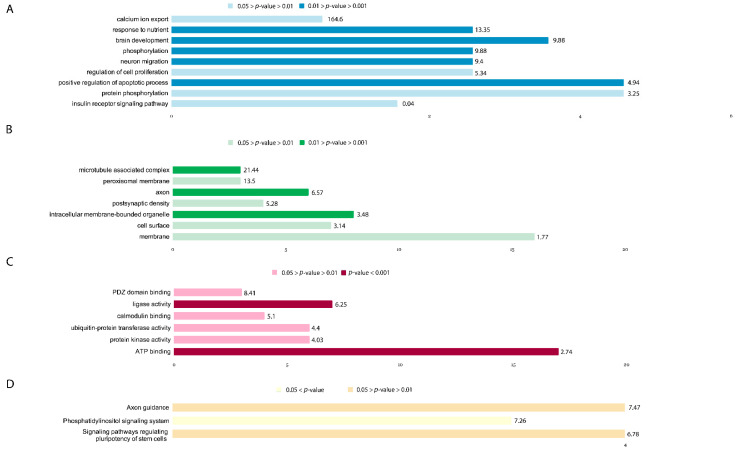
Downregulated GO groups by biological processes (**A**). Downregulated GO groups by cellular components (**B**). Downregulated GO groups by molecular function (**C**). Downregulated gene groups by KEGG (**D**). Labels for each GO group are plotted on the *y*-axis, the number of genes present within the GO group—on the *x*-axis. Fold enrichment scores for the corresponding GO groups are presented at the end of each bar. Results within a given plot are color-coded by *p*-value.

Sixteen hours of incubation with 30 nM ouabain caused a 1.2-fold (*p* < 0.05) decrease in expression of 81 genes, which were sorted in the same way as upregulated genes: biological processes (GOTERM_BP_DIRECT)—15 groups (groups of interest shown in Figure 6A, Supplementary Table S5), cellular components (GOTERM_CC_DIRECT)—12 groups (groups of interest shown in Figure 6B, Supplementary Table S6), molecular function (GOTERM_MF_DIRECT)—7 groups (groups of interest shown in Figure 6C, Supplementary Table S7). Genes were also grouped in accordance with their involvement in metabolic pathways using the KEGG database (KEGG_PATHWAY)—4 groups (groups of interest shown in Figure 6D, Supplementary Table S8). The full lists of genes and GO groups can be found in Supplementary materials, sheet “DOWNreg genes”.

Groups containing downregulated genes associated with biological processes (Figure 6A) included the following: GO:0007584~response to nutrient (4 genes: HMGCR—3-Hydroxy-3-Methylglutaryl-CoA Reductase, SLC8A1—Sodium/Calcium Exchanger, ACSL3—Acyl-CoA Synthetase Long Chain Family Member 3, CNR1—Cannabinoid Receptor 1; *p* = 0.0032, Fold Enrichment = 13.35); GO:0043065~positive regulation of apoptotic process (6 genes: ARHGEF12—Rho Guanine Nucleotide Exchange Factor 12, CSRNP3—Cysteine And Serine Rich Nuclear Protein 3, KALRN—Kalirin RhoGEF Kinase, PRKDC—DNA-Dependent Protein Kinase Catalytic Subunit, TXNIP—Thioredoxin Interacting Protein, CNR1; *p* = 0.007, Fold Enrichment = 4.94); GO:0007420~brain development (5 genes: BMPR2—Bone Morphogenetic Protein Receptor Type 2, ATP2B1—ATPase Plasma Membrane Ca^2+^ Transporting 1, ACSL3, PRKDC, ROBO2—Roundabout Guidance Receptor 2; *p* = 0.00745, Fold Enrichment = 9.877); GO:0016310~phosphorylation (4 genes: PPIP5K2—Diphosphoinositol Pentakisphosphate Kinase 2, PANK3—Pantothenate Kinase 3, ETNK1—Ethanolamine Kinase 1, N4BP2—NEDD4 Binding Protein 2; *p* = 0.0074, Fold Enrichment = 9.877); GO:0001764~neuron migration (4 genes: PCM1—Pericentriolar Material 1 Protein, DCC—Netrin 1 Receptor, FZD3—Frizzled Class Receptor 3, DCX—Doublecortin; *p* = 0.0085, Fold Enrichment = 9.4); GO:1901660~calcium ion export (2 genes: ATP2B1, SLC8A1; *p* = 0.012, Fold Enrichment = 164.63); GO:0006468~protein phosphorylation (6 genes: BIRC6—BIR Repeat-Containing Ubiquitin-Conjugating Enzyme, SCYL2—SCY1 Like Pseudokinase 2, GMFB—Glia Maturation Factor Beta, SNRK—SNF Related Kinase, KALRN, PIK3C3—Phosphatidylinositol 3-Kinase Catalytic Subunit Type 3; *p* = 0.035, Fold Enrichment = 3.25); GO:0042127~regulation of cell proliferation (4 genes: BMPR2, BIRC6, TFRC—Transferrin Receptor, TXNIP; *p* = 0.038, Fold Enrichment = 5.34); GO:0008286~insulin receptor signaling pathway (3 genes: SOGA1—Suppressor Of Glucose, Autophagy Associated 1, PIK3R3—Phosphoinositide-3-Kinase Regulatory Subunit 3, PHIP—Pleckstrin Homology Domain Interacting Protein; *p* = 0.039, Fold Enrichment = 9.5). The full list of genes and GO groups can be found in Supplementary Table S5.

GO sorting DEGs downregulated in the presence of 30 nM ouabain according to cellular components (Figure 6B) yielded the following groups: GO:0030424~axon (6 genes: SACS—Sacsin Molecular Chaperone, DCC, FZD3, DST—Dystonin, EPHA4—Tyrosine-Protein Kinase Receptor SEK, CNR1; *p* = 0.002, Fold Enrichment = 6.57); GO:0043231~intracellular membrane-bounded organelle (8 genes: SACS, DCC, FZD3, DST, EPHA4, CNR1; *p* = 0.0074, Fold Enrichment = 3.48); GO:0005875~microtubule associated complex (3 genes: RANBP2—RAN Binding Protein 2, MAP2—Microtubule Associated Protein 2, DCX; *p* = 0.008, Fold Enrichment = 21.44); GO:0005778~peroxisomal membrane (3 genes: HMGCR, ACSL3, CNOT1—CCR4-NOT Transcription Complex Subunit 1; *p* = 0.02, Fold Enrichment = 13.5); GO:0009986~cell surface (7 genes: BMPR2, ADGRV1—Adhesion G Protein-Coupled Receptor V1, SLC1A2—Excitatory Amino Acid Transporter 2, FZD3, TFRC, EPHA4, ROBO2; *p* = 0.023, Fold Enrichment = 3.14); GO:0016020~membrane (16 genes: RANBP2, ATP2B1, SLC1A2, PRKDC, TFRC, MIA3—MIA SH3 Domain ER Export Factor 3, PIK3C3, DENND5B—DENN Domain-Containing Protein 5B, ARHGEF12, ADGRV1, PCM1, ETNK1, SLC8A1, ACSL3, CNOT1; *p* = 0.029, Fold Enrichment = 1.767); GO:0014069~postsynaptic density (4 genes: BMPR2, KALRN, MIB1, EPHA4; *p* = 0.039, Fold Enrichment = 5.28). The full list of genes and GO groups can be found in Supplementary Table S6.

DEGs downregulated in the presence of 30 nM ouabain into GO groups by molecular function (Figure 6C) can be found in Supplementary Table S7. Among them are the following: GO:0005524~ATP binding (17 genes: CHD9, ATP2B1, PRKDC, EPHA4, N4BP2, PIK3C3, HSPA13, BMPR2, PPIP5K2, PANK3, SCYL2, ETNK1, SNRK, ACSL3, KALRN, SMCHD1, SHPRH; *p* < 0.001, Fold Enrichment = 2.74); GO:0016874~ligase activity (7 genes: BIRC6, RANBP2, DZIP3, ACSL3, MIB1, SHPRH, HECTD2; *p* < 0.001, Fold Enrichment = 6.25); GO:0004842~ubiquitin-protein transferase activity (6 genesBIRC6, DZIP3, PHOSPHO2-KLHL23, MIB1, SHPRH, HECTD2; *p* = 0.011, Fold Enrichment = 4.4); GO:0004672~protein kinase activity (6 genes: SCYL2, SNRK, KALRN, PRKDC, EPHA4, PIK3C3; *p* = 0.0156, Fold Enrichment = 4.03); GO:0005516~calmodulin binding (4 genes: ATP2B1, MAP2, SLC8A1, DCX; *p* = 0.042, Fold Enrichment = 5.1); GO:0030165~PDZ domain binding (3 genes: ATP2B1, CXXC4, FZD3; *p* = 0.048, Fold Enrichment = 8.41).

Sorting DEGs downregulated in the presence of 30 nM ouabain by KEGG pathway (Figure 6D) yielded the following groups: hsa04360: Axon guidance (4 genes: ARHGEF12, DCC, EPHA4, ROBO2; *p* = 0.014, Fold Enrichment = 7.47); hsa04550:Signaling pathways regulating pluripotency of stem cells (4 genes: BMPR2, PIK3R3, RIF1—Replication Timing Regulatory Factor 1, FZD3; *p* = 0.019, Fold Enrichment = 6.78); hsa04070:Phosphatidylinositol signaling system (3 genes: PPIP5K2, PIK3R3, PIK3C3; *p* = 0.06, Fold Enrichment = 7.26). The full list of genes and GO groups can be found in Supplementary Table S8.

### 3.8. Description of the Main Gene Clusters

**Figure 7:**
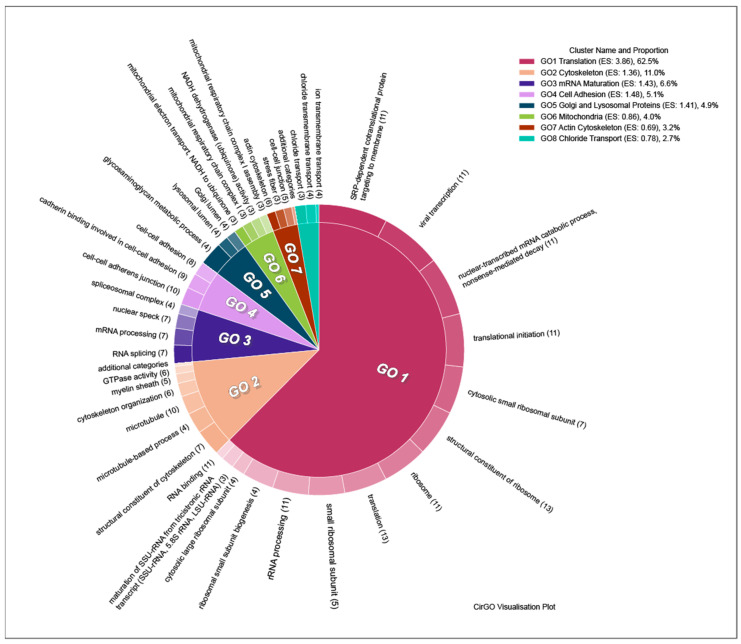
Clusters and GO groups representing genes the expression of which was upregulated after culture incubation with ouabain. Image generated using CirGO software [44]. The inner circle contains the main clusters, while the outer represents the GO groups subordinate to each cluster. Slice size is determined by the absolute log10 *p*-value of GO groups, the smallest *p*-value corresponding to the largest slice. Color gradients are used to emphasize the largest to smallest value distribution in the outer ring sub-groups. ES: Enrichment Score.

Using DAVID clusterization, groups of DEGs were sorted into eight clusters for upregulated genes, and five clusters for downregulated genes. The most representative cluster for the upregulated genes is the ribosome-associated translation cluster, which includes genes from both ribosomal elements and the translation process as a whole (Enrichment Score: 3.86). This cluster includes 14 groups of genes. Genes associated with microtubule cytoskeleton organization, especially that of tubulin, were also found to be upregulated. This cluster includes six gene groups (Enrichment Score: 1.36). Upregulated genes were also sorted into clusters associated with cellular adhesion (Enrichment Score: 1.48), mRNA maturation (Enrichment Score: 1.43), Cl^−^ transport through the membrane (Enrichment Score: 0.78), mitochondria (Enrichment Score: 0.86), and Golgi and lysosomal proteins (Enrichment Score: 1.41) (Figure 7). The complete list of clusters and groups of genes included in them can be found in Supplementary materials, sheet “DOWNreg Clusters”.

**Figure 8:**
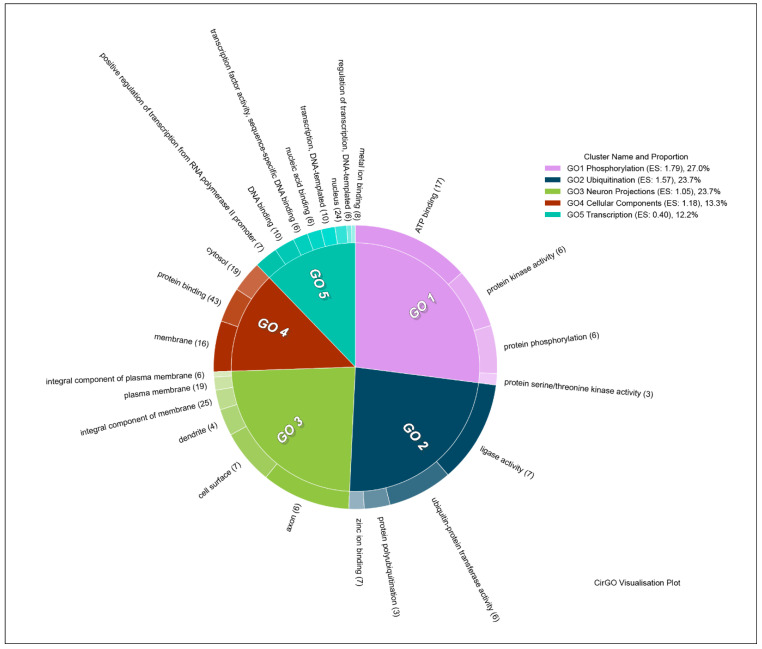
Clusters and GO groups representing genes the expression of which was downregulated after culture incubation with ouabain. Image generated using CirGO software [44]. The inner circle contains the main clusters, while the outer represents the GO groups subordinate to each cluster. Slice size is determined by the absolute log10 *p*-value of GO groups, the smallest *p*-value corresponding to the largest slice. Color gradients are used to emphasize the largest to smallest value distribution in the outer ring sub-groups. ES: Enrichment Score.

Due to the smaller number of downregulated genes, clusterization analysis was less informative than that for the upregulated genes. Downregulated genes were sorted into five clusters. These included clusters represented by genes associated with phosphorylation (Enrichment Score: 1.79), and ubiquitination (Enrichment Score: 1.57), neuron projections (Enrichment Score: 1.05), miscellaneous cellular components (Enrichment Score: 1.18), and transcription (Enrichment Score: 0.40) (Figure 8). The full list of clusters and included gene groups can be found in Supplementary materials, sheet “UPreg Clusters”.

### 3.9. Predicted Transcription Factors Which Can Regulate the Expression of Identified DEGs

Since we were interested in identifying the signaling cascades associated with ouabain-induced changes in DEG expression, we used MAGIC software to conduct an upstream analysis of the acquired gene lists with an ouabain-induced fold change of 1.2 or greater. 175 potential transcription factors were identified for the upregulated genes, and 70 potential transcription factors for down regulated genes. The full list of factors can be found in Supplementary materials, sheet “Transcription Factors”.

## 4. Discussion

Results of human iPSC-derived neuron culture immunocytochemical analysis show that the culture in question expresses markers characteristic of GABAergic neurons, expressing DARPP-32 and, potentially, receiving dopaminergic input. A similar approach to confirming neuronal differentiation via staining for beta-tubulin, MAP2, DARPP-32, GFAP, and TH has been used in other studies [48,49]. As mentioned previously, to investigate the influence of ouabain on gene expression in human iPSC-derived neurons, we used the maximum dose of non-toxic ouabain. We did not find any studies demonstrating the toxicity of ouabain for human iPSC-derived neurons. However, for human iPSC-derived cardiomyocytes the minimal toxic ouabain concentration is 100 nM, which is in accordance with our data [50]. It is known that ouabain in the concentrations investigated induces changes in the Na^+^/K^+^ ratio in the cytoplasm of human cells by inhibiting the Na^+^,K^+^-ATPase [51]. Furthermore, it is known that increased intracellular Na^+^ concentration in neurons leads to an increase in Ca^2+^ concentration [12]. In turn, changes in intracellular ion concentration lead to activation of various signaling pathways [52]. Previous studies have shown that ouabain, together with changes in intracellular ion concentrations, induces CREB activation [53,54]. This effect of ouabain in concentrations of 30 nM and 300 nM was shown in the present study. Our data also showed that ouabain in a concentration of 30 nM or greater causes ERK1/2 activation. The findings are in line with multiple studies conducted previously both on neurons and other cell types [29,55]. Therefore, we used 30 nM ouabain to evaluate changes in gene expression, since it is a non-toxic concentration which induces changes in signaling pathway activation in cultured cells.

The observed increase in expression of genes associated with ribosomes, translation initiation, and mRNA maturation allows us to conclude that ouabain induces increased protein synthesis in neurons. This conclusion is in accordance with prior research showing the influence of ouabain on protein synthesis [56], as well as data showing the influence of ouabain on gene expression in a primary rat cerebellum neuron culture [53].

It is known that increased expression of ribosomal genes is necessary for neuron development [57]. Maintenance of protein translation levels as a whole is considered key to neuron vitality, and decreased translation levels accompany the development of neurodegenerative pathologies [58]. As such, the observed ouabain-induced increase in expression of genes associated with initiation and elongation of translation speaks to a positive influence on neuron maturation and their vitality.

It is logical that during maturation, neurons are constantly restructuring and expanding their cytoskeleton, and require large amounts of energy generated by the mitochondria. We have shown that incubation with ouabain leads to increased expression of genes associated with tubulin and actin cytoskeleton organization, as well as vesicular transport and phagocytosis. Previously, it has been suggested that ouabain can induce cytoskeleton remodeling [59]. Our results build on this finding, and allow for a fuller understanding of the complete set of genes the expression of which mediates this effect of ouabain. Ouabain also induces increased expression of a cluster of genes associated with cellular adhesion, including regulators of metalloproteases, caveolar proteins, and cytoskeleton adapter proteins. An increase in expression of microtubule tubulin cytoskeleton genes, which is necessary for organization of the neurite cytoskeleton [60] should also be noted. Increased expression of JIP-2 and JIP-3, which interacts with JNK, has also been observed. The expression of these two genes positively correlates with the speed of JNK-mediated axon lengthening [61,62]. These proteins are also necessary for NMDA-mediated signal transduction [63]. This data contributes to our understanding of the mechanisms behind the observed effects of ouabain on intercellular junctions [64], together with the recently published study describing ouabain-induced phosphoproteome changes [65]. As such, increased expression of genes associated with neurite growth and cytoskeleton organization can be noted. On the other hand, a 20% decrease in MAP2 and Dystonin, which are necessary for neuron cytoskeleton organization [66,67], can be observed. As a whole, it can be concluded that ouabain induces changes in expression of genes associated with neural cytoskeleton dynamics and organization, but further investigations are required to elucidate the full picture.

Moreover, ouabain induces increased expression of genes associated with the synthesis of synaptic structural proteins and myelin sheath formation. For example, increased expression of Agrin [68], Calsyntenin-3 (CST-3) [69], ELFN1 [70], and PIN1 [71] is observed. These genes are necessary for synaptogenesis and functional organization of the synapse. Another effect of ouabain relates to the expression of genes associated with glycosaminoglycan synthesis. This relationship between ouabain and glycosaminoglycans has not been previously observed in neurons, but it was shown in cartilage tissue that ouabain induces an increase in extracellular matrix durability via increased intracellular Na^+^ and Ca^2+^ concentrations [72].

It is known that glycosaminoglycans play an important role in neurite growth, synaptic plasticity, and regeneration in the CNS. Impairment of their synthesis is linked to CNS pathologies [73]. For example, increased expression of the serine-threonine kinases BRSK1 and BRSK2 (SAD1), which regulates neuron polarization, synapse formation, and neurotransmitter release from synaptic vesicles [74,75,76]. ITM2C expression is associated with synapse formation and decreased GABAergic transmission, while its disruption has been linked to Alzheimer’s disease [77,78].

One of the effects mediated by ouabain is an increased expression of components of the mitochondrial electron transport chain (ETC)—for example, the NADH-dehydrogenase. This data gives ground to better understanding of the mechanisms underlying the ouabain-induced increase in mitochondrial ETC activity observed in the rat ouabain-induced mania model. Perhaps the ouabain-induced increase in mitochondrial ETC activity is also associated with increased neurite formation, since it is known to occur during the development of neural junctions and synaptogenesis [79].

Of special interest is the increased expression of cyclin-dependent kinase inhibitors CDKN1C and CDKN1A, which inhibit proliferation [80], and BMP7, which is associated with presynaptic retrograde signaling and neuroregeneration [81]. On the contrary, decreased expression of gene groups associated with proliferation, early CNS development, neuron migration, and positive apoptosis regulation is observed.

As such, it can be concluded that the presence of ouabain influences gene expression in such a way as to suppress proliferation and assist neuron maturation. This is in line with previous studies, in which ouabain in a concentration of 0.1–1 uM induced a significant CREB-dependent increase in neurite growth speed in a rat brain cortex neuron primary culture [82].

This data may, possibly, shed light on the mechanisms behind ouabain’s apparent effects in enhancing functional CNS recovery demonstrated in the mouse brain trauma model [83]. Furthermore, it is known that restoration of dopaminergic transmission is vital in recuperation after an insult [84], while GABAergic projections into the substantia nigra are important in maintaining dopaminergic transmission [84].

Increased expression of genes associated with Cl^−^ transport, such as SLC4A3, has also been observed. It has earlier been shown that ouabain can increase CFTR-mediated Cl^−^ efflux [85]. At the same time, a decrease in expression of Ca^2+^ transporters, the Plasma Membrane Calcium-Transporting ATPase 1 (PMCA1) and the Na^+^/Ca^2+^ Exchanger (NCX1). There is a plethora of data describing the functional interaction between the Na^+^,K^+^-ATPase and the NCX1 [12], even though a previous study has shown that 10 nM ouabain, on the contrary, leads to an increase in NCX1 protein in human arterial myocytes [86]. At the same time, there is no data addressing the functional interaction between the Na^+^,K^+^-ATPase and the PMCA1. Furthermore, it was shown that the Na^+^,K^+^-ATPase is not co-localized with PMCA1, unlike NCX1 [87]. Perhaps more attention should be directed to the potential interactions between the Na^+^,K^+^-ATPase and PMCA1 at the level of gene expression in further research, since PMCA2, like NCX1, is an important regulator of cytoplasmic Ca^2+^ concentration.

It should also be noted that ouabain induced decreased expression of a series of signaling kinase genes—including Diphosphoinositol Pentakisphosphate Kinase 2 (PPIP5K2), Ethanolamine Kinase 1 (ETNK1), Pantothenate Kinase 3 (PANK3), Kalirin RhoGEF Kinase (KALRN), Phosphatidylinositol 3-Kinase Catalytic Subunit Type 3 (PIK3C3), and Phosphoinositide-3-Kinase Regulatory Subunit 3 (PIK3R3)—which regulate an array of physiologically important neural processes. Components of the phosphoinositide signaling system play a key role in synaptic plasticity [88]. It was shown previously that the Na^+^,K^+^-ATPase interacts with IP3R [89], while ouabain causes PI3K activation [90]. Our results may promote understanding of these interactions as influenced by ouabain on the level of gene expression. On the other hand, we have shown that ouabain leads to increased expression of ERK1/2, MLK2 (MAPKKK), and p38β. MAPK (mitogen activated protein kinases) activity is closely associated both with prenatal and postnatal CNS development, and with functionality, vitality, and stress and apoptosis in mature neurons [29,91,92,93,94]. As such, it is impossible to draw any conclusions based on changes in expression of the abovementioned kinases in our experiments, due to the multiplicity of their possible effects in neurons based on a variety of different factors.

Analysis of proteins which can bond to promoters of DEGs uncovered in the study yielded a large number of universal transcription factors. Of special interest are the JUN and CREB families of proteins. The expression of JUND, for example, increased in the presence of ouabain. This is in line with previous studies of the influence of ouabain on gene expression in HUVEC cultures [37], as well as primary culture of cerebellum neurons [53] using GeneChip. However, any further assumptions addressing the relationship between transcription factors are listed in Supplementary Materials, sheet “Transcription Factors”, are bound to be speculative in nature. As such, it can only be said that this analysis may be used to fuel hypotheses for further research, which would allow for the construction of the complete picture of the effect of CTS in the CNS, but cannot be interpreted as fact.

**Figure 9:**
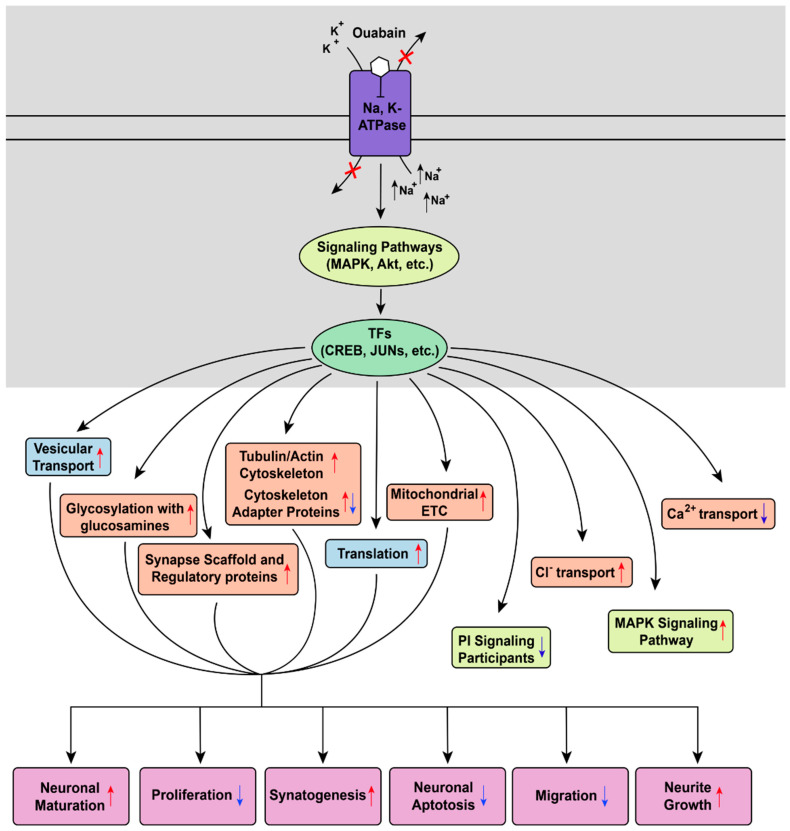
Diagram of the main processes induced by 16-h incubation of human iPSC-derived expressing DARPP-32 and GABA receptors neurons expressing DARPP-32 with 30 nM ouabain. Kinases and signaling pathways colored light green, transcription factors (TF) colored green. Cellular components and specific processes colored orange. Biological processes colored light purple. Up and down arrows signify increased and decreased gene expression, respectively.

In summary, a number of processes activated by 16 h incubation of human iPSC-derived expressing DARPP-32 and GABA receptors neurons, expressing the specific marker of dopamine-receptive neurons DARPP-32, with 30 nM ouabain can be singled out (Figure 9). We can conclude that the influence of ouabain leads to activation of genes responsible for neurite growth and synapse formation via increased expression of genes associated with translation, synapse formation, vesicular transport, and enhanced ETC function. At the same time, expression of genes associated with proliferation, migration, and early development of neurons is decreased. Taken together, these data indicate that ouabain application to DARPP-32 expressing GABA neurons induces neuron maturation, neurite growth, and increased synaptogenesis. Based on these effects, in turn, we conjecture that the influence of CTS in concentrations non-toxic to neurons may be linked to the establishment of new neuronal junctions and neuronal plasticity, in general. The observed changes in gene expression also suggest ouabain’s influence on Cl^−^ and Ca^2+^ ion transport, as well as the expression profile of key regulatory kinases. Finally, the data on changes in gene expression caused by ouabain may explain previously shown physiological effects facilitated by changes in the striatal dopaminergic and GABAergic transmission.

## Data Availability

All of the data is presented in the supplementary files. No additional data is reported.

## Supplementary Materials

The following are available online at https://www.mdpi.com/2076-3425/11/2/203/s1, Figure S1, Table S1: Upregulated GO groups by Biological Processes, Table S2. Upregulated GO groups by Cellular Components, Table S3. Upregulated GO groups by Molecular Function, Table S4. Upregulated gene groups by KEGG, Table S5. Downregulated GO groups by Biological Processes, Table S6. Downregulated GO groups by Cellular Components, Table S7. Downregulated GO groups by Molecular Function, Table S8. Downregulated gene groups by KEGG.

## Abbreviations

Akt	RAC-Alpha Serine/Threonine-Protein Kinase, Protein Kinase B Alpha
AMPA	A-Amino-3-Hydroxy-5-Methyl-4-Isoxazolepropionic Acid
ATP	Adenosine Triphosphate
BSA	Bovine Serum Albumin
CFTR	Cystic Fibrosis Transmembrane Conductance Regulator
CREB	Camp Response Element-Binding Protein
CST-3	Calsyntenin-3
CTS	Cardiotonic Steroids
DAPI	4′,6-Diamidino-2-Phenylindole
DARPP-32	Dopamine and CAMP-Regulated Neuronal Phosphoprotein 32
DAT	Dopamine Transporter
DAVID	Database For Annotation, Visualization And Integrated Discovery
Degs	Differentially Expressed Genes
Egr-1	Early Growth Response Protein 1
Eif5	Eukaryotic Translation Initiation Factor 5
ERK	Extracellular Signal-Regulated Kinase
GABA	Gamma-Aminobutyric Acid
GFAP	Glial Fibrillary Acidic Protein
GLAST	Glutamate Aspartate Transporter
GLT-1	Glutamate Transporter 1
GO	Gene Ontology
GSK 3	Glycogen Synthase Kinase 3
GTP	Guanosine Triphosphate
HBSS	Hanks’ Balanced Salt Solution
HUVEC	Human Umbilical Vein Endothelial Cells
ICV	Intracerebroventricular
Inos	Inducible Nitric Oxide Synthase
IP3R	Inositol Trisphosphate Receptor
JIP-2	JNK- Interacting Protein—2, Or MAPK8IP2—Mitogen-Activated Protein Kinase 8 Interacting Protein 2
JIP-3	JNK-Interacting Protein—3, Or MAPK8IP3—Mitogen-Activated Protein Kinase 8 Interacting Protein 3
JNK	C-Jun N-Terminal Kinas
KEGG	Kyoto Encyclopedia Of Genes And Genomes
MAP2	Microtubule Associated Protein 2
MAPK	Mitogen-Activated Protein Kinases
MLK2	Mixed Lineage Kinase 2, Or MAPKKK—Mitogen-Activated Protein Kinase Kinase Kinase
MTT	3-(4,5-Dimethyl-2-Thiazolyl)-2,5-Diphenyl-2H-Tetrazolium Bromide
NCX	Sodium-Calcium Exchanger, Na^+^/Ca^2+^ Exchanger
NMDA	N-Methyl-D-Aspartate
PBS	Phosphate-Buffered Saline
PI3K	Phosphoinositide 3-Kinase
PKC	Protein Kinase C
PMCA1	Plasma Membrane Calcium-Transporting Atpase 1
PMCA2	Plasma Membrane Calcium-Transporting Atpase 2
PPIP5K2	Diphosphoinositol Pentakisphosphate Kinase 2
PVDF	Polyvinylidene Difluoride
RIN	RNA Integrity Number
RIPA Buffer	Radioimmunoprecipitation Assay Buffer
SDS-PAGE	Sodium Dodecyl Sulphate–Polyacrylamide Gel Electrophoresis
SLC4A3	Solute Carrier Family 4 Member 3
Th	Tyrosine Hydroxylase
VCAM-1	Vascular Cell Adhesion Molecule 1
VSMC	Vascular Smooth Muscle Cells
