# Supplementary material for: Ouabain-Induced Gene Expression Changes in Human iPSC-Derived Neuron Culture Expressing Dopamine and cAMP-Regulated Phosphoprotein 32 and GABA Receptors
Source: Brain Sci. 2021 Feb 7;11(2):203. doi: 10.3390/brainsci11020203 (PMC7915459; doi:10.3390/brainsci11020203)
Supplement: Supplementary file 1 [file brainsci-11-00203-s001.zip › Lopachev et al Supplementary.docx]

Ouabain-Induced Gene Expression Changes in Human
iPSC-Derived Neuron Culture Expressing Dopamine and
cAMP-Regulated Phosphoprotein 32 and GABA Receptors

Alexander V. Lopachev ^1,^*, Maria A. Lagarkova ^2^, Olga S. Lebedeva ^2^, M.A. Ezhova ^3,4^,
Rogneda B. Kazanskaya ^5^, Yulia A. Timoshina ^1,6^, Anastasiya V. Khutorova ^1,6^, Evgeny E. Akkuratov ^7^,
Tatiana N. Fedorova ^1^, and Raul R. Gainetdinov ^8^

Laboratory of Clinical and Experimental Neurochemistry, Research Center of Neurology, 125367 Moscow, Russia; july.timoschina@yandex.ru (Y.A.T.); hutorova.anastasiya@mail.ru (A.V.K.); tnf51@bk.ru (T.N.F.)

^2^ Laboratory of Cell Biology, Federal Research and Clinical Center of Physical-Chemical Medicine Ofederal Medical Biological Agency, 119435Moscow, Russia; lagar@rcpcm.org (M.A.L.); oslebedeva@rcpcm.org (O.S.L.)

^3^ Laboratory of Plant Genomics, Institute for Information Transmission Problems of the Russian Academy of Sciences, 12705 Moscow, Russia; a.fedotova@skoltech.ru

^4^ Center of Life Sciences, Skolkovo Institute of Science and Technology, 121205 Moscow, Russia

^5^ Biological Department, Saint Petersburg State University, 199034 St. Petersburg, Russia;
st059046@student.spbu.ru

^6^ Biological Department, Lomonosov Moscow State University, 119991 Moscow, Russia

^7^ Department of Applied Physics, Royal Institute of Technology, Science for Life Laboratory,
171 65 Stockholm, Sweden; akkuratov.evgeny@gmail.com

^8^ Institute of Translational Biomedicine and Saint Petersburg University Hospital, Saint Petersburg State
University, 199034 St Petersburg, Russia; gainetdinov.raul@gmail.com

Expression of the α3 and α1 isoform of the Na^+^,K^+^-ATPase α-subunit

One important characteristic of neurons is the expression of the α3 isoform of the Na^+^,K^+^-ATPase α-subunit. We analyzed protein lysates of both the intact (control) culture and the cultures which were incubated for four hours with 3 nM, 30 nM, and 300 nM ouabain to determine the presence of α3 and α1 isoforms of the Na^+^,K^+^-ATPase α subunit using Western Blot.


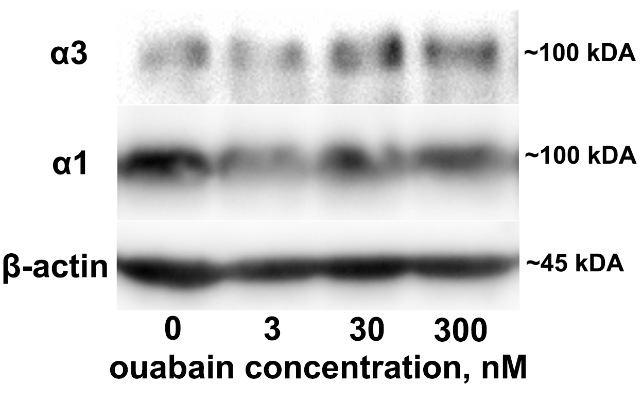


**Figure S1.** Immunoreactive bands representing the α3 and α1 isoforms of the Na^+^,K^+^-ATPase α-subunit, as well as β-actin for reference.

As seen in Figure 2, the human iPSC-derived neuron culture used in these experiments expresses both α3 and α1 isoforms of the Na^+^,K^+^-ATPase α-subunit. 4 h incubation with 3 nM, 30 nM, and 300 nM ouabain does not noticeably affect the expression of either of these proteins.

Description of the main GO groups containing upregulated genes

As mentioned previously, 256 genes, the expression of which significantly (padj < 0.05) increased more than 1.2 fold in comparison to the control culture after a 16 h incubation with 30 nM ouabain, were selected for further analysis. The selected genes were sorted into gene ontology (GO) groups in accordance with the three ontologies: Biological processes (GOTERM_BP_DIRECT) - 44 groups (Groups of interest shown in Table 1), cellular components (GOTERM_CC_DIRECT) - 29 groups (Table 2), molecular function (GOTERM_MF_DIRECT) - 20 groups (Groups of interest shown in Table 3). Genes were also grouped in accordance with their involvement in metabolic pathways using the KEGG database (KEGG_PATHWAY) - 7 groups (Groups of interest shown in Table 4). Two genes associated with synapse organization also should be mentioned separately: AGRN - Agrin Proteoglycan, and ELFN1 - Extracellular Leucine Rich Repeat And Fibronectin Type III Domain Containing 1. The full lists of genes and GO groups can be found in Supplementary materials, sheet “UPreg genes”.

**Table S1.** Upregulated GO groups by Biological Processes.

| **GO Accession Number~GO Term** | **Gene Count** | **Gene Symbols** | ***p*-Value** | **Fold  Enrichment** |
| --- | --- | --- | --- | --- |
| GO:0006614~SRP-dependent cotranslational protein targeting to membrane, GO:0006413~translational initiation | 11 | FAU, RPL23A, RPS9, RPS24, RPS16, RPL30, RPL18, RPS19, RPS15, RPL13, RPS11 | 2.51E-07, 8.01E-06 | 9.4, 6.45 |
| GO:0006412~translation | 13 | RPL23A, RPS24, RPS15, FAU, RPS9, SLC25A6, RPS16, RPL30, MRPL41, RPL18, RPS19, RPL13, RPS11 | 7.63E-05 | 4.13 |
| GO:0006364~rRNA processing | 11 | FAU, RPL23A, RPS9, RPS24, RPS16, RPL30, RPL18, RPS19, RPS15, RPL13, RPS11 | 3.44E-04 | 4.13 |
| GO:0042274~ribosomal small subunit biogenesis | 4 | RPS24, RPS16, RPS19, RPS15 | 9.31E-04 | 20.09 |
| GO:0006491~N-glycan processing | 4 | MGAT4B, PRKCSH, MAN2A2, MAN1B1 | 0.00183 | 16.07 |
| GO:0030203~glycosaminoglycan metabolic process | 4 | B3GALT6, AGRN, GPC1, BCAN | 0.0054 | 11.08 |
| GO:0007017~microtubule-based process | 4 | TUBB2B, TUBB4A, TUBB4B, TUBB2A | 0.0099 | 8.93 |
| GO:0000302~response to reactive oxygen species | 4 | PTPRN, PRDX5, GSTP1, GPX1 | 0.01234 | 8.24 |
| GO:0008380~RNA splicing | 7 | SCAF1, PUF60, SNRNP70, SF3A2, SNRPD2, AKAP8L, AKAP17A | 0.01738 | 3.39 |
| GO:0051591~response to cAMP | 4 | BSG, VGF, JUND, PTPRN | 0.01924 | 6.99 |
| GO:0006397~mRNA processing | 7 | SCAF1, PUF60, SNRNP70, SF3A2, AKAP8L, AKAP17A, AURKAIP1 | 0.02419461703 | 3.14 |
| GO:0006915~apoptotic process | 14 | PUF60, TRAF4, MFSD10, FKBP8, MAP3K10, PIDD1, SCAND1, SQSTM1, ADAM15, CST3, SLC25A6, MRPL41, PRDX5, MAPK3 | 0.02455 | 1.98 |
| GO:0042326~negative regulation of phosphorylation | 3 | CDKN1C, BMP7, CDKN1A | 0.02506 | 12.05 |
| GO:0032355~response to estradiol | 5 | CST3, MMP15, IGFBP2, GSTP1, BMP7 | 0.02651 | 4.41 |
| GO:0030182~neuron differentiation | 5 | BRSK2, ITM2C, BRSK1, PIGT, PIN1 | 0.03041 | 4.23 |
| GO:0002486~antigen processing and presentation of endogenous peptide antigen via MHC class I via ER pathway, TAP-independent | 2 | HLA-C, HLA-A | 0.0367 | 53.56 |
| GO:0006977~DNA damage response, signal transduction by p53 class mediator resulting in cell cycle arrest | 4 | PCBP4, PIDD1, PRMT1, CDKN1A | 0.04157 | 5.18 |
| GO:0006974~cellular response to DNA damage stimulus | 7 | PIDD1, OTUB1, TAOK3, BRSK1, MAPK3, CDKN1A, ALKBH7 | 0.04518 | 2.7 |
| GO:0060393~regulation of pathway-restricted SMAD protein phosphorylation | 2 | BMP7, PIN1 | 0.04864 | 40.17 |

**Table S2.** Upregulated GO groups by Cellular Components.

| **GO Accession Number~GO Term** | **Gene Count** | **Gene Symbols** | ***p*-Value** | **Fold Enrichment** |
| --- | --- | --- | --- | --- |
| GO:0031012~extracellular matrix | 17 | FGFBP3, PLEC, CRIP2, VIM, FLNA, COL6A1, SLC25A6, AGRN, RPS16, RPL30, MMP15, TUBB4B, LTBP4, RPS19, BMP7, ADAMTS10, RPS11 | 1.06E-06 | 4.57 |
| GO:0070062~extracellular exosome | 63 | see Supplementary for full list | 3.30E-06 | 1.78 |
| GO:0022627~cytosolic small ribosomal subunit | 7 | FAU, RPS9, RPS24, RPS16, RPS19, RPS15, RPS11 | 2.84E-05 | 11.6 |
| GO:0005840~ribosome | 11 | FAU, RPL23A, RPS9, RPS24, RPS16, RPL30, RPL18, RPS19, RPS15, RPL13, RPS11 | 4.68E-05 | 5.27 |
| GO:0005925~focal adhesion | 16 | BSG, FLOT2, ACTN4, PLEC, CD81, VIM, RPS15, PDLIM7, FLNA, RPS9, RPS16, RPL30, RPL18, RPS19, MAPK3, RPS11 | 1.27E-04 | 3.26 |
| GO:0016020~membrane | 47 | see Supplementary for full list | 3.00E-04 | 1.7 |
| GO:0015935~small ribosomal subunit | 5 | FAU, RPS9, RPS24, RPS16, RPS15 | 3.34E-04 | 14.74 |
| GO:0005829~cytosol | 60 | see Supplementary for full list | 0.0023 | 1.44 |
| GO:0005765~lysosomal membrane | 11 | COL6A1, SLC44A2, CD63, AP3D1, TMEM175, ITM2C, SPNS1, GAA, TMEM8A, SPPL2B, ABCA2 | 0.0024 | 3.19 |
| GO:0005743~mitochondrial inner membrane | 13 | ATP5MG, SPNS1, NDUFB7, AURKAIP1, NDUFS7, NDUFS5, MRPS26, SLC25A6, MRPL41, NME4, CYC1, ACADVL, TIMM44 | 0.0098 | 2.35 |
| GO:0005874~microtubule | 10 | TUBB2B, MTA1, TUBB4A, TUBB4B, KIFC2, TUBB2A, TBCB, TUBGCP6, CDK5RAP3, CAMSAP3 | 0.0167 | 2.56 |
| GO:0005913~cell-cell adherens junction | 10 | BSG, FLNA, FLOT2, PUF60, ALDOA, RPL23A, SLC3A2, PLEC, FSCN1, H1FX | 0.021 | 2.46 |
| GO:0005686~U2 snRNP | 3 | SF3B5, SF3A2, SNRPD2 | 0.0255 | 11.94 |
| GO:0005737~cytoplasm | 79 | see Supplementary for full list | 0.038 | 1.2 |
| GO:0071011~precatalytic spliceosome | 3 | SF3B5, SNRNP70, SNRPD2 | 0.0387 | 9.55 |
| GO:0016607~nuclear speck | 7 | RING1, SNRNP70, CBX4, SF3A2, AKAP8L, AKAP17A, PIN1 | 0.041 | 2.77 |

**Table S3.** Upregulated GO groups by Molecular Function.

| **GO Accession Number~GO Term** | **Gene Count** | **Gene Symbols** | ***p*-Value** | **Fold  Enrichment** |
| --- | --- | --- | --- | --- |
| GO:0044822~poly(A) RNA binding | 35 | see Supplementary for full list | 2.91E-06 | 2.4 |
| GO:0003735~structural constituent of ribosome | 13 | RPL23A, RPS24, RPS15, FAU, RPS9, SLC25A6, RPS16, RPL30, MRPL41, RPL18, RPS19, RPL13, RPS11 | 3.12E-05 | 4.53 |
| GO:0016301~kinase activity | 12 | BRSK2, CKB, OBSL1, CDKN1C, MAPK8IP3, AKAP8L, TAOK3, PRKCSH, MAPK3, CDKN1A, AURKAIP1, DGKZ | 2.97E-04 | 3.86 |
| GO:0005200~structural constituent of cytoskeleton | 7 | TUBB2B, TUBB4A, AGRN, TUBB4B, VIM, TUBB2A, TUBGCP6 | 0.00297 | 4.93 |
| GO:0042802~identical protein binding | 18 | PUF60, TRAF4, SDF4, PSMA7, RABAC1, FKBP8, PFKL, SSNA1, SRM, DRAP1, VIM, SCAND1, PAFAH1B3, ALDOA, SQSTM1, CST3, PRMT1, FTL | 0.01679 | 1.86 |
| GO:0004602~glutathione peroxidase activity | 3 | GPX4, GSTP1, GPX1 | 0.02942 | 11.06 |
| GO:0031625~ubiquitin protein ligase binding | 9 | TRAF4, CDC34, SQSTM1, CKB, UBE2M, OTUB1, ANAPC2, UBXN1, CDKN1A | 0.03232 | 2.43 |
| GO:0098641~cadherin binding involved in cell-cell adhesion | 9 | BSG, FLNA, PUF60, ALDOA, RPL23A, SLC3A2, PLEC, FSCN1, H1-10 | 0.03409 | 2.4 |
| GO:0017134~fibroblast growth factor binding | 3 | FGFBP3, GPC1, RPS19 | 0.03486 | 10.1 |
| GO:0046977~TAP binding | 2 | HLA-C, HLA-A | 0.03807 | 51.62 |
| GO:0042605~peptide antigen binding | 3 | HLA-C, HLA-A, SLC7A5 | 0.04996 | 8.29 |

**Table S4.** Upregulated gene groups by KEGG.

| **KEGG Accession Number/ Term** | **Count** | **Genes** | ***p-*Value** | **Fold  Enrichment** |
| --- | --- | --- | --- | --- |
| hsa03010:Ribosome | 11 | FAU, RPL23A, RPS9, RPS24, RPS16, RPL30, RPL18, RPS19, RPS15, RPL13, RPS11 | 4.16E-05 | 5.2 |
| hsa04145:Phagosome | 7 | HLA-C, ATP6V0B, TUBB2B, HLA-A, TUBB4A, TUBB4B, TUBB2A | 0.02812 | 3 |
| hsa00480:Glutathione metabolism | 4 | GPX4, SRM, GSTP1, GPX1 | 0.04301 | 5.04 |
| hsa04540:Gap junction | 5 | TUBB2B, TUBB4A, TUBB4B, TUBB2A, MAPK3 | 0.0462 | 3.65 |
| hsa01100:Metabolic pathways | 27 | ISYNA1, SRM, INPP5E, ATP5MG, PNPLA2, MAN2A2, DGKZ, ATP6V0B, PAFAH1B3, PIGQ, NDUFS5, MVD, PIGT, ACADVL, GUK1, B3GALT6, PFKL, GAA, NDUFB7, MAN1B1, NDUFS7, ALDOA, CKB, PTGES2, MGAT4B, NME4, CYC1 | 0.0472 | 1.42 |
| hsa00190:Oxidative phosphorylation | 6 | ATP6V0B, NDUFS7, NDUFS5, ATP5MG, NDUFB7, CYC1 | 0.05431 | 2.9 |

Description of the main GO groups containing downregulated genes

16 h of incubation with 30 nM ouabain caused an 1.2 fold (padj < 0.05) decrease in expression of 81 genes, which were sorted in the same way as upregulated genes: Biological processes (GOTERM_BP_DIRECT) - 15 groups (Groups of interest shown in Table 5), cellular components (GOTERM_CC_DIRECT) - 12 groups (Groups of interest shown in Table 6), molecular function (GOTERM_MF_DIRECT) - 7 groups (Groups of interest shown in Table 7). Genes were also grouped in accordance with their involvement in metabolic pathways using the KEGG database (KEGG_PATHWAY) - 4 groups (Groups of interest shown in Table 8). The full lists of genes and GO groups can be found in Supplementary materials, sheet “DOWNreg genes”.

**Table S5.** Downregulated GO groups by Biological Processes.

| **GEO Accession Number~GO Term** | **Gene Count** | **Gene Symbols** | ***p*-Value** | **Fold  Enrichment** |
| --- | --- | --- | --- | --- |
| GO:0007584~response to nutrient | 4 | HMGCR, SLC8A1, ACSL3, CNR1 | 0.0032 | 13.35 |
| GO:0043065~positive regulation of apoptotic process | 6 | ARHGEF12, CSRNP3, KALRN, PRKDC, TXNIP, CNR1 | 0.007 | 4.94 |
| GO:0007420~brain development | 5 | BMPR2, ATP2B1, ACSL3, PRKDC, ROBO2 | 0.00745 | 9.877 |
| GO:0016310~phosphorylation | 4 | PPIP5K2, PANK3, ETNK1, N4BP2 | 0.0074 | 9.877 |
| GO:0001764~neuron migration | 4 | PCM1, DCC, FZD3, DCX | 0.0085 | 9.4 |
| GO:1901660~calcium ion export | 2 | ATP2B1, SLC8A1 | 0.012 | 164.63 |
| GO:0006468~protein phosphorylation | 6 | BIRC6, SCYL2, GMFB, SNRK, KALRN, PIK3C3 | 0.035 | 3.25 |
| GO:0042127~regulation of cell proliferation | 4 | BMPR2, BIRC6, TFRC, TXNIP | 0.038 | 5.34 |
| GO:0008286~insulin receptor signaling pathway | 3 | SOGA1, PIK3R3, PHIP | 0.039 | 0.039 |

**Table S6.** Downregulated GO groups by Cellular Components.

| **GO Accession Number~GO Term** | **Gene Count** | **Gene Symbols** | ***p*-Value** | **Fold  Enrichment** |
| --- | --- | --- | --- | --- |
| GO:0030424~axon | 6 | SACS, DCC, FZD3, DST, EPHA4, CNR1 | 0.002 | 6.57 |
| GO:0043231~intracellular membrane-bounded organelle | 8 | SACS, DCC, FZD3, DST, EPHA4, CNR1 | 0.0074 | 3.48 |
| GO:0005875~microtubule associated complex | 3 | RANBP2, MAP2, DCX | 0.008 | 21.44 |
| GO:0005778~peroxisomal membrane | 3 | HMGCR, ACSL3, CNOT1 | 0.02 | 13.5 |
| GO:0009986~cell surface | 7 | BMPR2, ADGRV1, SLC1A2, FZD3, TFRC, EPHA4, ROBO2 | 0.023 | 3.14 |
| GO:0016020~membrane | 16 | RANBP2, ATP2B1, SLC1A2, PRKDC, TFRC, MIA3, PIK3C3, DENND5B, ARHGEF12, ADGRV1, PCM1, ETNK1, SLC8A1, ACSL3, CNOT1 | 0.029 | 1.767 |
| GO:0014069~postsynaptic density | 4 | BMPR2, KALRN, MIB1, EPHA4 | 0.039 | 5.28 |

**Table S7.** Downregulated GO groups by Molecular Function.

| **GO Accession Number~GO Term** | **Gene Count** | **Gene Symbols** | ***p*-Value** | **Fold  Enrichment** |
| --- | --- | --- | --- | --- |
| GO:0005524~ATP binding | 17 | CHD9, ATP2B1, PRKDC, EPHA4, N4BP2, PIK3C3, HSPA13, BMPR2, PPIP5K2, PANK3, SCYL2, ETNK1, SNRK, ACSL3, KALRN, SMCHD1, SHPRH | 2.75E-04 | 2.74 |
| GO:0016874~ligase activity | 7 | BIRC6, RANBP2, DZIP3, ACSL3, MIB1, SHPRH, HECTD2 | 8.17E-04 | 6.25 |
| GO:0004842~ubiquitin-protein transferase activity | 6 | BIRC6, DZIP3, PHOSPHO2-KLHL23, MIB1, SHPRH, HECTD2 | 0.01109 | 4.4 |
| GO:0004672~protein kinase activity | 6 | SCYL2, SNRK, KALRN, PRKDC, EPHA4, PIK3C3 | 0.01566 | 4.03 |
| GO:0005516~calmodulin binding | 4 | ATP2B1, MAP2, SLC8A1, DCX | 0.04225 | 5.1 |
| GO:0030165~PDZ domain binding | 3 | ATP2B1, CXXC4, FZD3 | 0.0483 | 8.41 |

**Table S8.** Downregulated gene groups by KEGG.

| **KEGG Accession Number/ Term** | **Count** | **Genes** | ***p-*Value** | **Fold  Enrichment** |
| --- | --- | --- | --- | --- |
| hsa04360: Axon guidance | 4 | ARHGEF12, DCC, EPHA4, ROBO2 | 0.014 | 7.47 |
| hsa04550:Signaling pathways regulating pluripotency of stem cells | 4 | BMPR2, PIK3R3, RIF1, FZD3 | 0.019 | 6.78 |
| hsa04070:Phosphatidylinositol signaling system | 3 | PPIP5K2, PIK3R3, PIK3C3 | 0.06 | 7.26 |
