# Supplementary figures and images for: Ouabain-Induced Gene Expression Changes in Human iPSC-Derived Neuron Culture Expressing Dopamine and cAMP-Regulated Phosphoprotein 32 and GABA Receptors
Source: Brain Sci. 2021 Feb 7;11(2):203. doi: 10.3390/brainsci11020203 (PMC7915459; doi:10.3390/brainsci11020203)

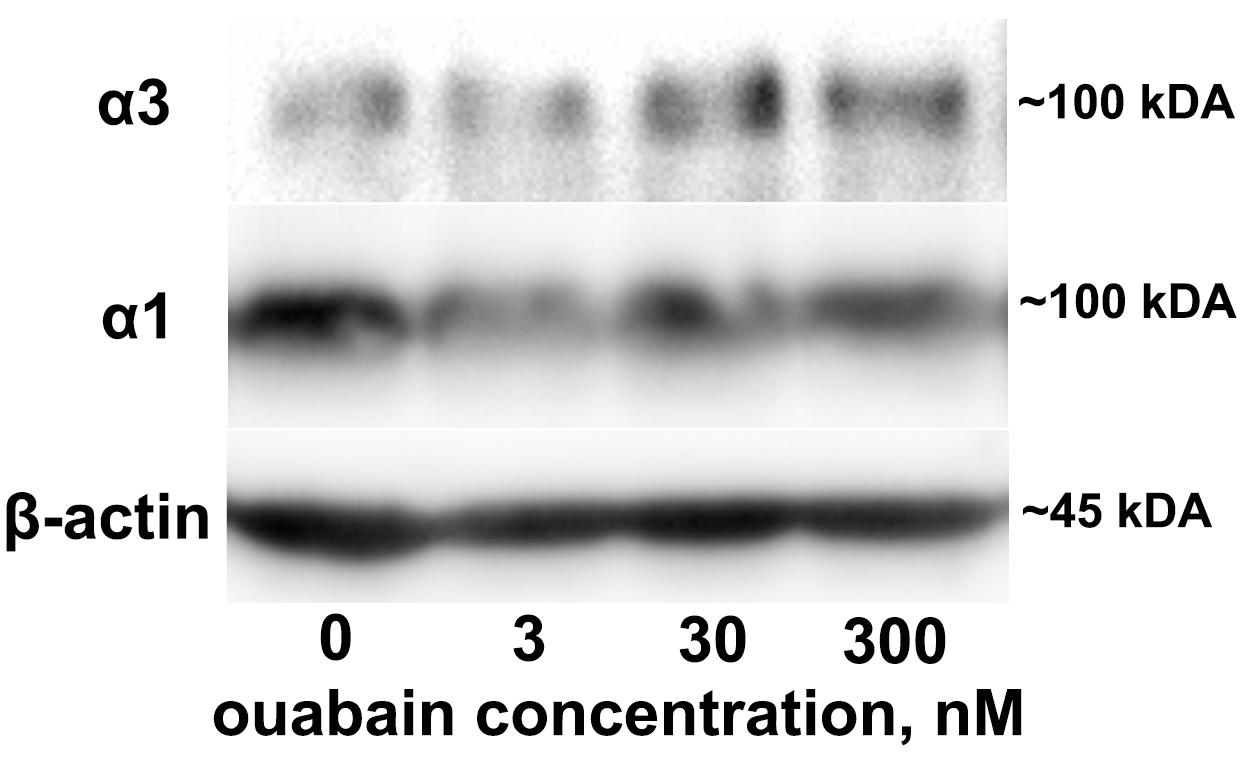

Supplement: Supplementary file 1 [file brainsci-11-00203-s001.zip › Figure 1S.tif]
